# Safety of esophagogastroduodenoscopy-guided forceps biopsy and the feasibility of esophagogastroduodenoscopy for evaluation of hypopharyngeal cancer

**DOI:** 10.1186/s12893-019-0571-z

**Published:** 2019-08-08

**Authors:** Hyun Jun Hong, Seok-Hoo Jeong, Won Shik Kim, Yu Jin Kim

**Affiliations:** 1 Department of Gastroenterology, International St. Mary’s Hospital, Catholic Kwandong Universtiy College of Medicine, Incheon, South Korea; 2 Department of Otorhinolaryngology, International St. Mary’s Hospital, Catholic Kwandong Universtiy College of Medicine, Incheon, South Korea; 3 Division of Gastroenterology, Deparment of Internal Medicine, Kangnam Sacred-Heart Hospital, Hallym University Medical Center, Hallym University College of Medicine, Seoul, South Korea; 4 Division of Gastroenterology, Department of Internal Medicine, Kangnam Sacred-Heart Hospital, Hallym University Medical Center, Hallym University College of Medicine, Chuncheon, South Korea; 5 Yonsei University College of Medicine, 1, Singil-ro, Yeongdeungpo-gu, Seoul, Republic of Korea

**Keywords:** Esophagogastroduodenoscopy, Forceps biopsy, Hypopharyngeal cancer

## Abstract

**Background:**

There is currently no established standard tissue sampling method for hypopharyngeal cancer. The present study aimed to evaluate the feasibility of esophagogastroduodenoscopy (EGD) for the pretreatment evaluation of hypopharyngeal cancer and the safety of EGD-guided forceps biopsy.

**Methods:**

We reviewed nine patients with hypopharyngeal cancer who underwent EGD for the evaluation of tumor extent and tissue biopsy from March 2014 to March 2017 at International St. Mary’s Hospital. One experienced endoscopist performed all the EGD procedures in the presence of a head and neck surgeon. The procedure included determining tumor location, extent (presence of pyriform sinus apex involvement), and size, and passing the endoscope through the upper esophageal sphincter. The success rate of tissue sampling was assessed, and procedure-related complications were recorded.

**Results:**

All patients were male, with a mean age of 69.9 ± 10.9 years (range 61–69 years). Tissue sampling using biopsy forceps was performed in 6/9 patients (66.7%). No complications related to moderate sedation or biopsy, including post-biopsy bleeding or respiratory distress, were reported. Histologic confirmation was successful in 5/6 patients (83.3%). Upper gastrointestinal lesions were evaluated in 7/9 (77.8%) patients in whom the scope passed through the lesion.

**Conclusions:**

EGD and EGD-guided forceps biopsy may be useful for the evaluation of hypopharyngeal cancer extent and tissue sampling, respectively.

**Electronic supplementary material:**

The online version of this article (10.1186/s12893-019-0571-z) contains supplementary material, which is available to authorized users.

## Background

Hypopharyngeal cancer accounts for approximately 5% of all head and neck cancers [1, 2]. Anatomically, the hypopharynx extends from the plane of the hyoid bone above to the plane of the inferior border of the cricoid cartilage below. Hypopharyngeal cancer usually does not cause symptoms until late in the disease course [3] and has a higher incidence of early metastasis and poorer prognosis than laryngeal cancer [4].

Hypopharyngeal cancer is relatively uncommon and anatomically complex. Various treatment options have been used based on its stage [5]. The results of radiotherapy alone are comparable to those of partial surgery for early hypopharyngeal cancer [6]. Surgical resection, followed by radiotherapy, if necessary, reportedly results in a better survival rate in patients with advanced cancers [7, 8]. However, the potential consequences of a radical surgical approach include significant alterations in voice and swallowing function, or complete loss of one or both. Complications such as fistulas and stenosis are common, and patients may require further surgery if they survive [5]. Therefore, it is important to select the best treatment option.

Pretreatment diagnosis includes histologic confirmation and staging. Although squamous cell carcinoma comprises more than 90% of cancers of the hypopharynx [9], histological confirmation of the tumor is essential. Direct visualization of the lesion using a rigid laryngoscope under general anesthesia and tissue sampling is currently the standard diagnostic method. Patients with hypopharyngeal cancer are usually of old age and present with medical comorbidities. Therefore, a surgical procedure under general anesthesia is a burden for both the patient and the surgeon.

Upper gastrointestinal endoscopes have some advantages over rigid or flexible laryngoscopes, as they have better resolution and flexibility and can detect concomitant esophageal squamous dysplasia or carcinoma. Wang et al. [10] described the evaluation of upper gastrointestinal non-neoplastic lesions using an ultrathin endoscope. Due to the risk of bleeding and airway obstruction, however, tissue sampling using a gastroscope is not the procedure of choice. As of yet, no standard diagnostic test has been established. The present study aimed to evaluate the feasibility of gastroscopy for the evaluation of tumor extent, and the safety of tissue sampling using a gastroscope.

## Methods

### Patients

The medical records of consecutive primary hypopharyngeal cancer patients who underwent esophagogastroduodenoscopy (EGD) from March 2014 to March 2017 at International St. Mary’s Hospital were retrospectively reviewed. Hypopharyngeal tumors were detected via neck computed tomography (CT), magnetic resonance imaging (MRI), and/or laryngoscopy before EGD. All study protocols were retrospectively approved by our institutional review board (submission number IS17RASI0065).

### EGD and tissue sampling

One endoscopist (Kim YJ) performed all EGD procedures in the presence of one head and neck surgeon (Hong HJ). A single-channel gastroscope (GIF-Q260J, Olympus, Tokyo, Japan) or an ultrathin endoscope (GIF-XP260N, Olympus, Tokyo, Japan) was used with or without sedation. Tissue sampling was performed using disposable biopsy forceps (FB-230 K, Olympus, Tokyo, Japan). Inspection, photography, and narrow-band imaging of hypopharyngeal lesions were performed both before and after passage through the pyriform sinus. During EGD, primary tumor extent, scope passage through the pyriform sinus, number of tissue samples taken using biopsy forceps, and the presence or absence of concomitant esophageal and gastric lesions were recorded. After EGD, we also assessed the accuracy of diagnosis based on EGD-guided biopsy histology and post-procedure complications, including post-biopsy hemorrhage or perforation. The accuracy of pathologic diagnosis using EGD guided forceps biopsy and passage of the endoscope through the tumor were evaluated.

## Results

Table 1 shows the demographic data and clinical staging of the nine patients. All patients were male, with a mean age of 69.9 ± 10.9 years (range 61–69 years). Five patients underwent concurrent chemoradiation therapy, and the other four were administered best palliative care.

**Table 1:** Demographic data and clinical stages of the enrolled patients

Patient number	Sex	Age range (years)	Stage	Treatment
00298963	male	65–70	cT2N0M0,	CCRT
00298607	male	60–65	cT4aN0Mx	cT4aN0Mx
00272563	male	60–65	cT4aN2cMx	CCRT
00164374	male	75–80	cT4aN1Mx	CCRT
00294335	male	85–90	cT4bN2cMx	best supportive care
00252398	male	50–55	cT2N2bMx	CCRT
00247856	male	80–85	cT4bN2bMx	best supportive care
00225664	male	60–65	–	best supportive care
00176506	male	75–80	cT4aN2cMx	best supportive care

*CCRT* concurrent chemoradiation therapy

**Fig. 1:**
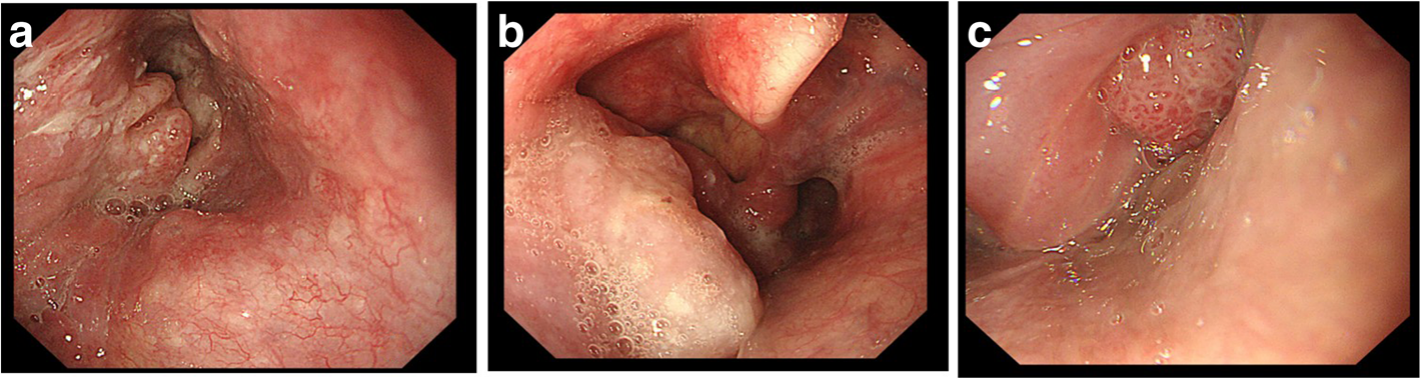
Hypopharyngeal cancers at different locations. **a** Pyriform sinus, **b** posterior pharyngeal wall, **c** post-cricoid area

Tumors were located in the pyriform sinus, post-cricoid area, and posterior pharyngeal wall (Fig. 1). The lesions were located in the pyriform sinus in 5/9 patients (Table 2). Endoscopy was observed in real time by one ear, nose, and throat specialist (Hong HJ) (Additional file 1: Video S1), who assessed tumor location and extent, as well as the involvement of the pyriform sinus inlet.

**Table 2:** Results of esophagogastroduodenoscopy in the enrolled patients

Patient number	Sex	Age range (years)	Sedation	Location	Histologic diagnosis using EGD forceps	Complication	Passage of scope	Number of biopsied tissue fragments	Concomitant upper gastrointestinal disease
00298963	male	65–70	+	posterior pharyngeal wall and pyriform sinus	–	no	yes	0	reflux esophagitis, gastric polyp, duodenal ulcer active stage
00298607	male	60–65	–	pyriform sinus	squamous cell carcinoma	no	yes	4	atrophic gastritis, intestinal metaplasia
00272563	male	60–65	–	pyriform sinus	squamous cell carcinoma	no	yes	3	duodenal polyp
00164374	male	75–80	+	pyriform sinus	squamous cell carcinoma	no	yes	3	atrophic gastritis, intestinal metaplasia
00294335	male	85–90	+	pyriform sinus	a few necrotic atypical squamous cells	no	no^a^	3	atrophic gastritis, intestinal metaplasia
00252398	male	50–55	+	postcricoid area	squamous cell carcinoma	no	yes	2	Gastric ulcer scar
00247856	male	80–85	+	posteriorpharyngeal wall	squamous cell carcinoma	no	yes	3	
00225664	male	60–65	–	posterior pharyngeal wall	–	no	yes	0	esophageal candidiasis
00176506	male	75–80	–	posterior pharyngeal wall/postcricoid area	–	no	no	0	

*EGD* esophagogastroduodenoscopy, ^a^Ultrathin scope passed through the pyriform sinus


**Additional file 1:** EGD-guided forceps biopsy for hypopharyngeal cancer. The gastroscope passed through the opposite side of the tumor to avoid bleeding. After observing the esopahgus, stomach and duodenum, the distal border of the tumor was assessed while withdrawing the scope. Tissue sampling was performed using biopsy forceps. (MP4 37513 kb)


**Fig. 2:**
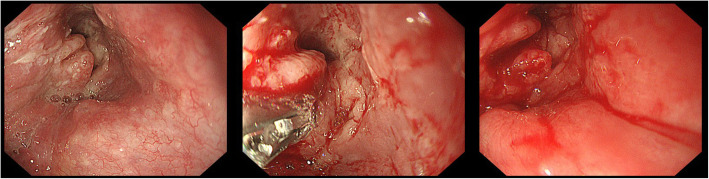
Tissue sample collection using biopsy forceps

EGD was performed with sedation in six patients and without sedation in three. The sedatives used were midazolam (0.07–0.15 mg/kg, intravenous) and pethidine (25–50 mg, intravenous). In two patients, the endoscope could not pass through the hypopharyngeal mass. In one patient, only an ultrathin endoscope could pass through the mass. Biopsy using forceps was performed in 6/9 patients (66.7%) (Fig. 2). In 3/9 patients, the role of EGD was confined to the evaluation of tumor extent. The success rate of forceps biopsy was 83.3% (5/6 patients). The number of tissue fragments ranged from two to four. The success of forceps biopsy was not significantly associated with the number of tissue fragments, location of the lesion, or use of sedation.

Procedures were performed on hospitalized patients. No complications related to moderate sedation or biopsy, including respiratory distress or post-biopsy bleeding, were reported. Concomitant upper gastrointestinal lesions included esophageal candidiasis, intestinal metaplasia, peptic ulcer scar, and duodenal polyp. No synchronous cancers were detected.

## Discussion

In the United States and Canada, 65–85% of hypopharyngeal carcinomas involve the pyriform sinuses, 10–20% involve the posterior pharyngeal wall, and 5–15% involve the post-cricoid area [11]. Due to its anatomical complexity, no standard treatment for cancer of the hypopharynx has been established. Expert guidelines recommend surgical resection for T1 cancer [12]. Expert guidelines recommend biopsy of the primary site or fine-needle aspiration of the neck, chest CT, CT with contrast, and/or MRI with contrast of the primary site and neck, and endoscopy under anesthesia for pretreatment evaluation [12]. To select surgical candidates, tumor extent should be evaluated. Rigid laryngoscopy under general anesthesia is usually the diagnostic method of choice.

Developments in endoscopy have made minimally invasive treatment possible in diseases of various organs. Flexible endoscopy has advantages over rigid laryngoscopy in that it is less uncomfortable for patients and does not require sedation. Gastrointestinal endoscopy yields better resolution than flexible laryngoscopy, and enables the concomitant evaluation of esophageal, gastric, and duodenal lesions.

Five studies have reported that office-based biopsy for head and neck cancers under local anesthesia using flexible digital video laryngoscopy or transnasal fiberoptic endoscopy is safe and can successfully yield a histopathological diagnosis [13–17]. Of these five studies, three included hypopharyngeal cancer patients (*n* = 2 a, *n* = 8 d *n* = 8), while the other two included only laryngeal cancer patients. Hypopharyngeal cancer extends below the pyriform sinus inlet, and sometimes lesions are not visible via laryngoscopy. Moreover, the incidence of synchronous esophageal dysplasia or carcinoma is higher in patients with hypopharyngeal cancer than in the general population, and upper gastrointestinal endoscopy is essential. Biopsy and upper gastrointestinal evaluation can be achieved using upper gastrointestinal endoscopes.

The success rate of tissue biopsy was 83.3% in the current study. In a previous study of hypopharyngeal cancer, the success rate was 100% [13]. The reported success rates of forceps biopsy through an endoscope biopsy channel in this and previous studies are acceptable; however, the currently available reported sample sizes are small.

No complications, including post-biopsy bleeding or respiratory distress during endoscopy, were observed in the present study. Mild to moderate sedation is needed, however, due to retching.

The study had some limitations. First, it was a single-center retrospective study, which may limit the reach of the conclusions; second, the study had a small sample size.

## Conclusions

EGD appears to be a feasible option for the pretreatment diagnosis and biopsy of hypopharyngeal cancer; our limited sample size showed that an acceptable success rate may be achieved. EGD can reduce the burden of general anesthesia and enables the concomitant evaluation of upper gastrointestinal lesions.

## Data Availability

The datasets used and/or analysed during the current study available from the corresponding author on reasonable request.

## Abbreviations

CT: Computed tomography
EGD: Esophagogastoduodenoscopy
MRI: Magnetic resonance imaging
